# Using PrEP and Doing it for Ourselves (UPDOs Protective Styles), a Web-Based Salon Intervention to Improve Uptake of Pre-exposure Prophylaxis Among Black Women: Protocol for a Pilot Feasibility Study

**DOI:** 10.2196/34556

**Published:** 2022-08-30

**Authors:** Schenita D Randolph, Ragan Johnson, Allison Johnson, Lana Keusch

**Affiliations:** 1 Duke University School of Nursing Duke University Durham, NC United States

**Keywords:** HIV prevention, women, PrEP uptake, application, web-based application, web-based, pre-exposure prophylaxis, prophylaxis, mixed-method, community engagement, pilot test, HIV

## Abstract

**Background:**

Multilevel interventions are necessary to address the complex social contributors to health that limit pre-exposure prophylaxis use among Black women, including medical distrust, pre-exposure prophylaxis stigma, and access to equitable health care. Strategies to improve knowledge, awareness, and uptake of pre-exposure prophylaxis among Black women will be more successful if information-sharing and implementation take place within trusted environments. Providing women with information through trusted cultural and social channels can effectively support informed decision-making about pre-exposure prophylaxis for themselves and members of their social networks who are eligible for pre-exposure prophylaxis.

**Objective:**

The goal of this project is to improve knowledge, awareness, uptake, and trust of pre-exposure prophylaxis, as well as reduce pre-exposure prophylaxis stigma, among Black women living in the US South.

**Methods:**

This multilevel, mixed methods study uses a community-engagement approach to develop and pilot test a salon-based intervention. There are three components of this intervention: (1) stylist training, (2) women-focused entertainment videos and modules, and (3) engagement of a pre-exposure prophylaxis navigator. First, stylist training will be provided through two 2-hour training sessions delivered over 2 consecutive weeks. We will use a pre- and posttest design to examine knowledge and awareness improvement of pre-exposure prophylaxis among the stylists. Upon full completion of training, the stylists will receive a certificate of completion and “Ask Me about PrEP” signage for their beauty salons. Second, together with the community, we have codeveloped a 4-part entertainment series (*The Wright Place*) that uses culturally and socially relevant stories to highlight key messages about (1) HIV, (2) pre-exposure prophylaxis, and (3) Black women’s social contributors to health. Quantitative and qualitative measures will be used in a pre- and posttest design to examine pre-exposure prophylaxis knowledge, awareness, risk, stigma, trust, intentions, and women’s perceptions of the usability and acceptability of the overall intervention and its implementation strategies. A video blog will be provided after each video. Third, participants will have access through an email or text message link to a pre-exposure prophylaxis navigator, who will respond to them privately to answer questions or make referrals for pre-exposure prophylaxis as requested.

**Results:**

This project was funded in October 2020 by Gilead Sciences and was approved by the Duke University School of Nursing institutional review board in April 2021 (Pro00106307). Intervention components were developed in partnership with community partners in the first year. Data collection for phase 1 began in April 2022. Data collection for phase 2 began in May 2022. The study will be complete by October 2022.

**Conclusions:**

Multilevel interventions that consider the assets of the community have promise for promoting health among Black women who have influence within their social networks. The findings of this study have the potential to be generalizable to other populations.

**International Registered Report Identifier (IRRID):**

PRR1-10.2196/34556

## Introduction

### Background

Pre-exposure prophylaxis (PrEP), taken as prescribed, is an effective HIV prevention strategy, yet uptake remains low among populations at risk for HIV in the US, especially Black women living in the US South [1]. Black women in the US comprise 13% of all women but account for over half (55%) of new HIV infections among women [2], and individuals living in the southern states are at the highest risk of acquiring HIV. Despite the critical need for interventions aimed at this population, no interventions that focus on Black women and PrEP are available in the *Compendium of Evidence-based Interventions and Best Practices for HIV Prevention,* published by the US Centers for Disease Control and Prevention (CDC) [3]. A small study of 10 Black women with sexual partners with HIV showed preliminary acceptability and feasibility of PrEP [4]; however, broad, multilevel interventions that reach Black women beyond those in serodiscordant relationships are needed.

### Barriers To Uptake

The barriers to PrEP uptake among Black women in the US are complicated, and individual- and interpersonal-level strategies, as well as community- and structural-level approaches, are warranted [5]. Barriers and challenges to PrEP uptake among Black women include lack of awareness and knowledge of PrEP [5-7], PrEP stigma [6,8], and distrust of medical professionals [6]. Yet interventions to increase PrEP have been (1) primarily focused on men who have sex with men (MSM), (2) implemented on the individual level, and (3) not been aimed at addressing PrEP stigma and medical distrust.

Lack of awareness and knowledge of PrEP among women in the US is prevalent [9,10]. Patel and colleagues [10] found that among 225 women living in the US South, 72% were eligible for PrEP; however, PrEP awareness was extremely low: only 11% of women had heard about PrEP. Signage campaigns such as “Ask Me about PrEP” have been used in clinical settings, but little research has linked their effectiveness to PrEP uptake among this population. To our knowledge, PrEP signage in community settings has not been examined and could be effective in reaching a broader population.

Stigma regarding PrEP is experienced personally among women, who also observe it within their social networks. Several studies show that women may not take PrEP due to fear that their family or friends will assume that the medication has been prescribed to treat HIV rather than prevent it [6-8]. These findings provide evidence that support from social networks plays a crucial role in determining women’s decision to start PrEP [10]. Strategies to reduce PrEP stigma, therefore, should leverage the social networks of Black women within trusted environments.

Finally, medical distrust is a significant barrier to health practices. Black women have been led by historic abuses to distrust the medical system [11], and this can affect their engagement with the health care system in general and with PrEP in particular [12,13]. For example, in a study of 500 female clients of Planned Parenthood living in 3 cities with high HIV prevalence, Black women had higher levels of medical distrust, evidenced by less comfort discussing PrEP with a medical provider [14]. One way to connect Black women with medical providers of PrEP is through linking a PrEP navigator to trusted and commonly frequented environments such as beauty salons. Patient navigators employed by local health departments can effectively decrease barriers faced by minority populations and increase completion of recommended health care utilization behaviors [15].

### Salon-Based Interventions

The feasibility and acceptability of interventions to encourage PrEP uptake and reduce barriers is of critical importance. Beauty salons and, in particular, stylists provide women with regular, trusted networks of influence and support; thus, they present unique opportunities to increase awareness of PrEP. The authors have previously shown that a salon-based intervention to promote awareness and uptake of PrEP would be feasible and acceptable among Black female salon customers (n=44), salon owners (n=6), and hair stylists (n=25) [16,17]. Salon-based research interventions have shown promise for promoting health broadly in Black communities, because stylists can share health information in the salon with Black women, who view them as trusted confidantes [16-21]. There is evidence that stylist and customer confidence is increased when stylists undertake training in preparation for sharing information [18], but only a single study conducted in Brazil has evaluated such a training program for beauty salon professionals [19]. Although women customers varied in their perceptions of the role of the stylists in health promotion in the salon setting, all stylists in our study reported the need to have current, factual health information to share with women as conversations arose.

Our previous work suggested that women customers would prefer the use of technology (ie, iPads, text messaging, email, and media) and culturally centered interventions for the delivery of health-related content in salons [16,17]. Technology-based approaches can target a larger number of individuals effectively at a lower cost and are conducive to addressing privacy concerns expressed in previous studies [16,17]. Specifically, the use of entertainment videos was an intervention strategy that was reported as being both culturally and socially relevant and engaging. Entertainment videos have been effective in improving HIV knowledge and HIV testing for women of color [22]. Furthermore, seeing other women who look like them may increase trust in health care systems and delivery.

### Aims and Objectives

**Figure 1:**
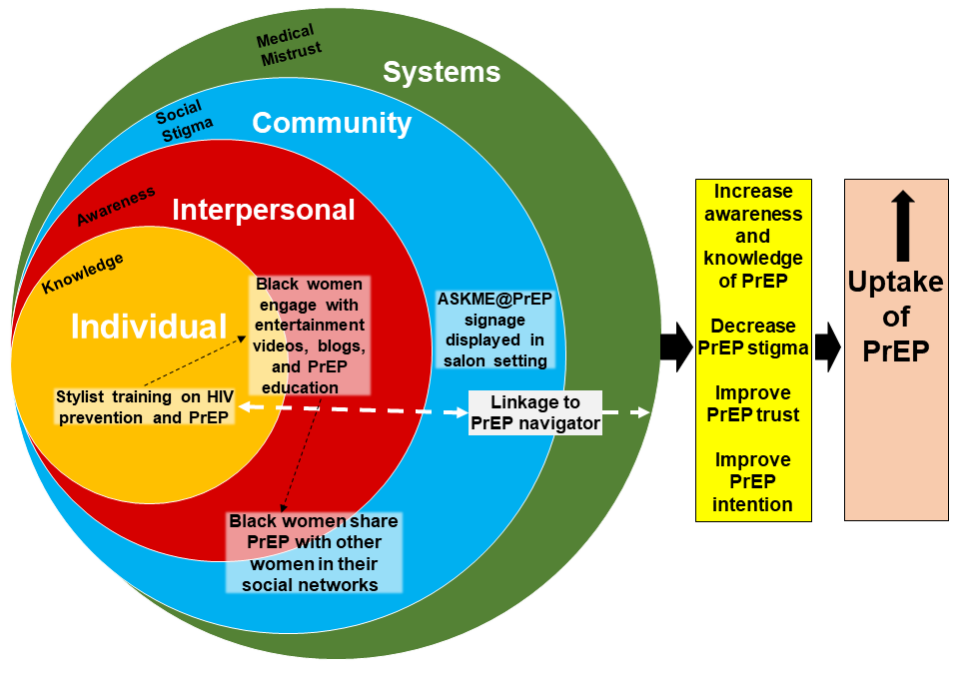
Socioecological model for the Using PrEP: Doing it for Ourselves (UPDOs) Protective Styles intervention. PrEP: pre-exposure prophylaxis.

The primary study objective was to develop a study protocol—Using PrEP: Doing it for Ourselves (UPDOs) Protective Styles—to test the feasibility and acceptability of a salon-based intervention to increase awareness, knowledge, and uptake of PrEP and reduce associated stigma and distrust of PrEP among Black women in the US South. UPDOs Protective Styles is a multiphase intervention that encompasses 3 sequential components (Figure 1). The first involves training stylists on women’s health and PrEP, virtually or face-to-face. The training protocol was adapted from the CDC’s evidence-based intervention “d-up: Defend Yourself!” [23], which enlists trusted community members whose advice is respected to serve as opinion leaders. D-up! is aimed at Black same-sex-loving men and uses opinion leaders to change social norms regarding condom use to prevent HIV [23]; our intervention protocol uses a similar approach. Stylists are respected and trusted in the Black community [16-19], and the training aims to leverage this respect to decrease social stigma associated with PrEP and HIV and increase trust in PrEP.

UPDOs’s second component entails entertainment-education, that is, videos designed to entertain while communicating prosocial norms and behaviors [24]. Videos have been used effectively to communicate HIV risk reduction and promote sexual health among Black women using smartphone-delivered, culturally relevant content, as has a stage play to increase awareness and knowledge of HIV produced for a Black community [25]. Both showed acceptability and feasibility and resulted in an increase in knowledge and awareness of HIV and prevention strategies. The videos developed for the current protocol each conclude with a video blog of the research team and community partners discussing its key points and providing resources, information, and links to related content. Community partners were cast members of the entertainment videos, stylists, or members of the community advisory committee. Blogs, in either written or video form, are often associated with popular-culture media and allow an opportunity to clarify, recap, or further explain concepts to the audience.

The final UPDO component is the integration of a local PrEP navigator as a resource for participants. Participants will have access to a recorded overview of the need for and role of PrEP navigators, as well as a digital link to their local navigator’s contact information.

### Theoretical Framework for Intervention

The design of this study was informed by the transtheoretical model [26], which posits that health behavior change involves progress through 6 stages of change: precontemplation, contemplation, preparation, action, maintenance, and termination [26]. Based on this model, the protocol-based study will evaluate women’s intentions to use PrEP at baseline, upon completion of the intervention, and at 3 and 6 months postintervention. Our multicomponent intervention aims to influence behavior change over time by engaging varied implementation strategies.

Two other frameworks have informed specific aspects of the protocol. Transportation theory [27] proposes that, to the extent that individuals are engaged in a story or “transported into a narrative world,” they may show effects of the story on their real-world beliefs and behaviors; this theory informed our development of the sitcom series that was videotaped for salon customers. Finally, a socioecological model [28] that considers the complex interplay between individual, relational, community, and other systems that influence one another guided development of the authors’ open-ended probes regarding the network- and health systems–related impact of the UPDOs intervention. These constructs may inform the sustainability of prevention efforts over time and the capacity within a community for longstanding reciprocal relationships that support health.

## Methods

### Design and Sample

#### Stylist Training

The training curriculum for stylists is based on d-up!, an evidence-based intervention developed by the CDC. This is a 4-part, 8-hour online learning module developed and facilitated by Black MSM, delivered to community leaders who can create or change social norms to promote condom use and address racial and sexual biases that increase risk for HIV [23]. D-up! has shown significant HIV-related positive outcomes and evidence of efficacy. For UPDOs, we integrated feedback from topic experts and a community advisory council of local stylists into a 2-part, 4-hour curriculum to be delivered face-to-face or by Zoom over 2 weeks. Part 1 includes information on HIV transmission, universal precautions for the stylist, social- and behavioral-level risks (eg, racism and bias) for HIV, and HIV prevention through PrEP. In part 2, stylists learn how to act as opinion leaders to change social norms regarding HIV and PrEP, including the opportunity for participants to practice conversations using case studies. Part 1 must be completed to progress to part 2 of the training. The curriculum is based on the most current evidence available from the CDC and the Black Women and PrEP Toolkit [29]. For reasons of confidentiality, sessions will not be recorded. Two facilitators will conduct each training session with a third team member who will serve as a notetaker.

A convenience sample of stylists in a 3-county catchment area of North Carolina will be recruited via word of mouth, flyers, social media, and active recruitment by study staff. Inclusion criteria include (1) full-time employment as a stylist or beauty industry professional, (2) age 18 years or older, (3) employment at a salon that serves primarily (≥50%) Black women customers, and (4) the ability to speak and understand English. All eligible and willing beauty industry professionals will be enrolled in the first study component in order to increase the number of opinion leaders who have accurate, evidence-based information. Stylists who complete the training are not required, as a part of this study, to have conversations with women customers about HIV and PrEP; such conversations are purely discretionary. Upon completion of their training, stylists will receive a certificate of completion and a QR code with signage (“Ask Me about PrEP”) to display in the salon. Finally, eligible stylists will be offered yearly 2-hour refresher training sessions on HIV prevention and PrEP.

Six salons that have completed the training curriculum will be selected to participate in the second component of the study. Stylists must be willing to display recruitment materials in the salon, serve as opinion leaders for their customers, and answer general questions about the study. Stylists who participate in the training will receive continuing education credit through the state’s Board of Cosmetology. Each of the 6 participating salons will receive US $500.

#### Entertainment-Education and Video Blogs

The videos are a series of four 20-minute sitcom episodes with accompanying blogs, titled *The Wright Place.* The series is socially and culturally relevant and was scripted by the study’s community partners with input from the research team, Black women, and the community advisory council; it was produced by a local filmmaker and executive producer. *The Wright Place* is grounded in evidence-based information from the CDC, the Black Women and PrEP Toolkit [29], and the extant literature. Each episode aims to improve Black women’s agency for their individual health and the health of those within their social networks. PrEP education is integrated with more general women’s health information (eg, cardiovascular disease and violence prevention) in the videos and blogs. Cardiovascular risk was chosen as it is the leading cause of death for Black women [30]. The videos and blogs are housed on a web-based platform accessible only to enrolled participants.

Black women will be recruited using rolling enrollment at the 6 salons among customers of stylists who agree to be opinion leaders and display the “Ask me about PrEP” signage in their salons, as well as through word of mouth, flyers, and efforts by research staff. Eligibility criteria include (1) age 18 years or older, (2) frequency of visiting the salon of at least every 2 weeks, (3) self-identification as Black or African American, (4) self-identification as a woman, and (5) ability to speak and read English. Based on their typical frequency of visiting the salon, women should expect to view 1 video and blog every 2 weeks. Participants must self-enroll in the study and will be able to access the videos and electronic surveys on their mobile device by typing in a link or using a QR code provided on flyers available in the salon. The QR code provides access to the consent form. After providing consent, participants will be sent a standardized welcome from the study team and given access to the web-based application link that they can access on their individual computers or smart devices. Thereafter, the study team communicates to the participants solely by email prompts or text messages, delivered from REDCap (Research Electronic Data Capture; Vanderbilt University) [31], to provide access to the web-based application link and administer survey measures. Stylists will not enroll participants nor administer study instruments. Compensation to Black women participants for their participation over 6 weeks will be US $125, which will be delivered in 2 payments.

After 60 women have completed the survey questionnaires and engaged in the site, a subsample of participants will be invited to join a single “think-aloud” focus group, with a goal of 10 women who have completed all parts of the intervention. Additionally, a focus group will be conducted with 6 stylists from the salons who have completed training and the full study. The purpose is to provide an opportunity to describe the experience of the UPDOs intervention and provide evaluative feedback to the study team. A lunch will be served at a site convenient to the group. Expected time needed for participation is 1.5 hours.

#### PrEP Navigator

**Figure 2:**
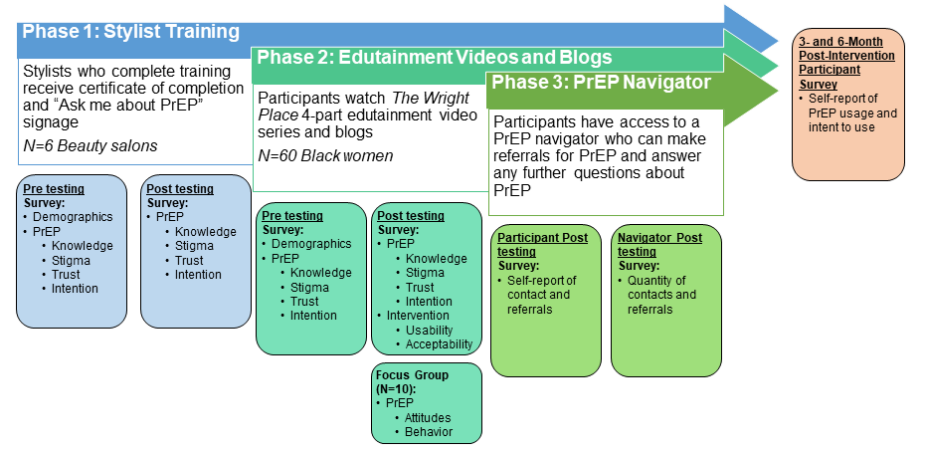
Study design schematic. PrEP: pre-exposure prophylaxis.

The final component of the study is a link to a PrEP navigator, an expert available through the state health department. There will be contact information on the web-based application to connect participants with a PrEP navigator; the participants themselves will initiate contact with the PrEP navigator, who will be available to discuss PrEP, answer PrEP-specific questions, and assist participants with obtaining an appointment at a PrEP clinic or beginning a PrEP conversation with their primary care provider or a provider of their choice. Through the web-based application, participants will have opportunities to share links to PrEP resources with members of their social networks through email or text notification; however, in the pilot testing phase, nonparticipants will not have open access to study materials (Figure 2).

### Measures

#### Stylists

A demographic questionnaire will be used to assess age, sexual orientation, marital status, zip code, history of sexually transmitted disease, and last HIV test for all participants. Each participant will have the opportunity to select more than one race and ethnicity upon screening and will be included if “Black” or “African American” are selected. HIV status will not be ascertained, in order to increase participation and avoid the potential for stigma.

Table 1 summarizes the measures used in this study. Two items, adapted from Chandler et al [32], will assess knowledge and awareness of PrEP: “Before this study, had you ever heard of PrEP?” (yes or no) and “On a range of 0 = no knowledge to 10 = expert knowledge, what is your knowledge of PrEP?” A further 31 items will be used to assess HIV and PrEP perceived risk (5 items), stigma (8 items), trust (12 items), and intentions (6 items). The perceived risk items are based on CDC guidelines [33]. Stigma will be assessed using 2 subscales (PrEP User Stereotypes and PrEP Disapproval by Others) of the PrEP Anticipated Stigma Scale [14]. Trust was assessed using Avanzo and colleagues’ [34] PrEP Trust Scale. Intention to use PrEP will be measured by responses to author-developed questions that are based on a transtheoretical model; for example, “I am not currently considering taking PrEP” (precontemplation) and “I am planning to take PrEP within the next month” (preparation). Responses range from 0 (very unlikely) to 5 (very likely). At 3 and 6 months postintervention, participants will self-report whether they have made and attended a clinic appointment for PrEP initiation, were prescribed PrEP, and utilized PrEP.

Feasibility and acceptability of the stylists’ curriculum will be measured with a 15-item pre- and posttest design survey to assess their comfort, willingness, and intention to serve as opinion leaders in their salon. A postquestionnaire will be delivered after the initial training and at the conclusion of the study to assess the acceptability of the content and format, including overall satisfaction with the training, willingness to participate in future training, and intention to share information learned in the training with Black women customers, other stylists, or members of the participant’s social networks. Open-ended questions on the postquestionnaire include (1) “Overall, how would you describe your experience with the opinion leader training?” (2) “What did you gain from this experience?” (3) “What were the best parts of the opinion leader training?” (4) “What were the least helpful or engaging parts of the training?” and (5) “How can the training be improved? Please provide specific recommendations when possible.”

**Table 1:** Summary of concepts and instruments used to collect the data.

Measure	Description	Expected outcome	Evaluation schedule
Systems Usability Scale [35]	A 10-item measure of digital intervention usability that allows evaluation of a wide variety of products and services, including hardware, software, mobile devices, websites and applications. Items are scored on a 5-item Likert scale, ranging from strongly disagree to strongly agree.	Assessment only	Postintervention
Acceptability and feasibility [22]	Video series/episodes	Assessment only	Postintervention
PrEP^a^ knowledge/awareness [32]	Two items: “Before this study, had you ever heard of PrEP?” (yes/no) and “On a range of 0 = no knowledge to 10 = expert knowledge, what is your knowledge of PrEP?”	Improved knowledge and awareness of PrEP	Baseline and postintervention
PrEP risk [33]	Five risk questions adapted from the CDC source documents: question 1 and questions 2-5 (yes/no response).	Assessment only	Baseline, postintervention
PrEP stigma [14]	Eight items; responses include “strongly disagree,” “disagree,” “agree,” and “strongly agree.” This measure assesses user stereotypes about PrEP and disapproval by others to take PrEP.	Improve stigma related to PrEP	Baseline, postintervention
PrEP trust [34]	Twelve items; responses are on a 10-point Likert scale ranging from “strongly disagree” to “strongly agree.” This measure perceptions about trust in the provider and health care interactions.	Improve trust related to PrEP	Baseline, postintervention
PrEP intentions [36]	Six items, including “How likely are you to use PrEP in the future?” Responses range from 0, “very unlikely,” to 5, “very likely.” This measure is based on the transtheoretical model of change.	Improve intentions of women to take PrEP	Baseline, postintervention, 3-6 months postintervention

^a^PrEP: pre-exposure prophylaxis.

#### Video/Blog Viewers

Survey items on participant demographic characteristics and barriers to PrEP uptake will be answered by viewers of the sitcom video and blogs using the same method as for the stylists. Although sexual risk is not an outcome of this study, the risk-related items will be included with the understanding that study participants who are not at significant risk themselves may know someone in their social network who is at risk. HIV status will not be ascertained, in order to increase participation and avoid the potential for stigma.

Acceptability and feasibility of the UPDOs intervention will be measured quantitatively using a 14-item evaluation tool (Multimedia Appendix 1**)** modified from a study by Jones et al [24]. Sample questions include “Do the episodes you watched address problems you think are important to women?” and “Do you think the episodes could help a woman make a decision about her sexual health?” Answers are ranked from “definitely no” to “definitely yes.” Feasibility data will also include the self-reported completion rate for all modules.

A focus group with a subsample of 10 women participants will be used to assess the implementation strategies and influence of the UPDOs intervention on the attitudes and behavior of the participants. Probes will address the usefulness for raising awareness and increasing uptake of PrEP of the following five aspects of UPDOs, as well as which aspects were most and least liked: (1) the “Ask me about PrEP” signage in salons, (2) the written section of the modules, (3) the entertainment-education videos, (4) the links and access to the PrEP navigator, and (5) impromptu conversations with stylists. To assess reach within social networks, participants will be asked if they shared information they learned about PrEP with their social networks, and if so, how often they shared it and whether they shared it with women, men, or both. The think-aloud protocol is an interview method designed to capture participants’ thought processes as they engage with instruments and interventions. The participant is asked to think aloud while solving a problem or completing a task, thus allowing understanding of their problem-solving process [37].

We will also evaluate the usability of the intervention website and application using the System Usability Scale (SUS) [35]. The SUS provides a reliable tool for measuring the usability of a wide variety of products and services, including websites and applications. It consists of a 10-item questionnaire with 5 response options for respondents, ranging from “strongly agree” to “strongly disagree.” Sample questions include “I think that I would like to use this system (website) frequently,” “I found the system (website) unnecessarily complex,” and “I thought the system (website) was easy to use.”

We will assess the proportion of participants who contact the PrEP navigator by self-report and the report of the PrEP navigator. These data will be deidentified. We have partnered with a local PrEP navigator for the development and implementation of the study protocol.

### Analytic Strategy

All assessments will be conducted using REDCap technology. Demographic information will be summarized as the mean and standard deviation for continuous variables and frequency and percentage for categorical variables.

Stylist training data will include training recruitment and completion rate as well as average time to complete surveys. Descriptive analyses will be conducted to describe pre- and posttraining PrEP awareness and knowledge and measures of barriers to PrEP uptake, and will be summarized as means, standard deviations, medians, interquartile ranges, and ranges. Given the small sample, a Wilcoxon test (or Wilcoxon signed-rank test) will be used to assess participant improvement in knowledge and awareness after completing the training. Descriptive statistics will be used to describe the feasibility and acceptability of UPDOs’s training for stylists. If improvement in awareness and knowledge is acceptable for an increase in scores of 2 standard units, we will be able to detect the desired change with 71% power for a sample of 4 and with 99.6% power with a sample of 8 [38].

The video/blog viewers will be assessed before and after completing their initial women’s health vignettes and modules on PrEP awareness, knowledge, and uptake. Awareness and knowledge total scores and the uptake of individual items will be descriptively summarized (as means, standard deviations, medians, interquartile ranges, and ranges), then tested using a paired *t* test. With a sample size of 60, for a 2-sided test with significance level set to *P*=.05, the paired *t* test will have 80% power to detect a mean standard difference of 0.37 [38]. Similarly, the number of women who received education from a stylist and the number of women who were referred to a PrEP navigator will be summarized descriptively.

Improvement in the 2 subscales of the PrEP Anticipated Stigma Scale (ie, PrEP User Stereotypes and PrEP Disapproval by Others) [14] and the PrEP Trust scale [34] (mean score of 12 items) will be graphically depicted over the multiple measurements (baseline, 3 months, and 6 months). A linear model for the longitudinal data will be fitted to estimate the linear improvement gained between baseline and each of the follow-up measurements. The unconditional models of the growth curve of the scores for the PrEP Anticipated Stigma PrEP User Stereotypes scale, the PrEP Anticipated Stigma Disapproval by Others scale, and the PrEP Trust scale have 80% power to detect a linear slope of 0.07 for 3-times measurements for a sample size of 60 [39]. Data collected on whether a woman customer saw a PrEP navigator or started PrEP, as well as on the number of referrals provided by women in the project, will be descriptively summarized.

The talk-aloud process and individual interviews will be audio-recorded, transcribed verbatim, and analyzed using directed content analysis [37]. We will analyze individual interviews separately using horizontalization, then develop clusters of meaning from significant statements by cross analysis to construct themes. We will perform systematic coding using a well-defined thematic codebook and NVivo data analysis software (QSR International). NVivo allows the coder to create a “tree” of codes that can be used to identify both individual and overlapping thematic units in the data. The research team will code the data separately and compare interpretations on an ongoing basis to achieve intercoder consensus and enhance the reliability and validity of the analyses. The resulting collection of themes will be conceptualized with the goal of creating an initial picture of the acceptability and usability of the salon-based intervention to increase PrEP awareness and uptake.

### Ethical Considerations

The study has been reviewed and was approved by the Duke University School of Nursing institutional review board in April 2021 (Pro00106307). The informed consent documents will include detailed information on all study procedures as well as the consent process. Stylists who attend the stylist training will provide electronic consent prior to training and before the start of the pretraining survey. Black women participants completing the video/blog intervention will provide electronic consent. Consent for the focus groups of women who will be engaged at the end of the pilot study will take place via verbal consent if COVID-19 prevents in-person meetings or via written consent if meetings are in person. In-salon discussions regarding PrEP and UPDOs are at the sole discretion of stylists and their customers; all assessments are opt-in opportunities and conducted on a digital device with whatever privacy settings are chosen by those being assessed.

## Results

This project was funded in October 2020 by Gilead Sciences, Inc and approved by the the Duke University School institutional review board in April 2021 (Pro00106307). Intervention components were developed in partnership with community partners in the first year. Data collection for the stylists’ training began in April 2022. Data collection for the video/blog viewers began in May 2022, and the study will be completed by October 2022. As of June 7, 2022, we have enrolled 3 salons, conducted stylist training with 4 stylists, and enrolled 17 women customers across the 3 salons.

## Discussion

### Anticipated Findings

This study will provide evidence that builds on the extant literature regarding whether and how partnering with trusted community members, such as salon stylists and PrEP navigators, may affect PrEP awareness, knowledge, uptake related to PrEP stigma, and medical trust among Black women living in the US South. Implementation of the UPDOs protocol will allow the authors to link their findings in a sample from a new population of Black women to evidence from prior studies among MSM and transgender women. This linkage, in addition to data regarding the feasibility and acceptability of the elements of the UPDOs protocol, may inform future intervention studies and, ultimately, the expansion of future CDC evidence–based interventions to include those specific to Black women, for whom there is a dearth of articles describing culturally appropriate interventions for HIV prevention. This gap is significant, given the size of this target population.

Detailing the lived experiences of Black women throughout the research process will allow evidence to be disseminated that could empower a community that displays significant health disparities [40] to have the information needed to make informed decisions for their own health. This level of engagement allows women to be in a position to share with other women and PrEP-eligible individuals in the community, thus potentially having community-wide impact.

### Strengths and Limitations

The design of this study has several strengths, including its focus on Black women, regardless of risk or HIV status, which has the potential to (1) reduce HIV- and PrEP-associated stigma and (2) uncover hidden risks, such as partner infidelity, that are beyond a woman’s control. The sample size and analysis by age and socioeconomic status have the potential to illuminate how information is shared among social networks, which are often intergenerational in the Black community. Lived experiences of Black women contributing to this work offer unique insights into the influences of where women work, live, and play. The influence of Black women in the family and community is well documented [41]. This study allows women to be leaders of this information, regardless of their perceived or actual vulnerability. Leveraging trusted spaces and networks of women offers a unique opportunity to change social norms around HIV prevention and the use of PrEP. If UPDOs demonstrates acceptability and feasibility in this pilot study, this approach has potential to have a broad impact on Black women’s health and the design of culturally relevant interventions.

The use of a web-based technology that participants can use from their personal device allows intervention testing with a potentially global reach while maintaining privacy, which can be sacrificed in interpersonal settings. An intervention that uses entertainment videos highlighting the social determinants of health for women is relatively novel and allows for a culturally relevant and timely impact on population health that could improve inequities among Black women in health and HIV prevention. Finally, the inclusion of training for stylists is critical, as prior research confirms it is necessary to ensure the comfort of stylists and Black women customers engaged in salon-based interventions. This education will empower stylists with requisite knowledge, as well as critical thinking and communication skills, to foster healthy sexual behaviors and positively influence social norms.

Anticipated limitations of this intervention include the self-reported PrEP uptake, as Black women may visit their primary care providers or other sources to receive PrEP. There is also potential for reporting bias on the initial assessment of PrEP risk; however, this information is not intended to inform the intervention. We also do not assess participants’ current HIV status in this study. Finally, the absence of a comparison group may limit the ability to determine whether change was related to the intervention or other factors.

### Future Directions

We have developed a strategic dissemination plan in partnership with members of our community advisory council, beauty industry partners, and a hired communication strategist’s team. Through our social media outlets, YouTube channel, short clip videos, a community forum led by salon partners, and quarterly newsletters, the research process and study findings will be shared with the community. Additionally, findings will be disseminated in peer-reviewed journals and at academic conferences.

## Appendix

Multimedia Appendix 1 Video Series/Episodes: The Wright Place from UPDOs Protective Styles Project.

## Abbreviations

CDC: US Centers for Disease Control and Prevention
d-up!: d-up: Defend Yourself!
HIV: human immunodeficiency virus
MSM: men who have sex with men
PrEP: pre-exposure prophylaxis
UPDOs: Using PrEP, Doing it for Ourselves
