# Supplementary material for: Using PrEP and Doing it for Ourselves (UPDOs Protective Styles), a Web-Based Salon Intervention to Improve Uptake of Pre-exposure Prophylaxis Among Black Women: Protocol for a Pilot Feasibility Study
Source: JMIR Res Protoc. 2022 Aug 30;11(8):e34556. doi: 10.2196/34556 (PMC9472057; doi:10.2196/34556)
Supplement: Multimedia Appendix 1 [file resprot_v11i8e34556_app1.pdf]

Video Series/Episodes: *The Wright Place* from UPDOs Protective Styles Project

Definitely No Don't Think so Maybe Probably Definitely Yes

1. Do the episodes you watched address problems you think are important to women?
2. Do you think the episodes could help a woman make decisions about her sexual health?
3. Do you think that watching the episodes could help raise a woman's awareness about her sexual health choices?
4. Were the stories realistic?
5. Do you know anyone who has gone through experiences similar to any of the lead characters?
6. Could the episodes you watched change a woman's stigma she may have around using PrEP? (STIGMA)
7. Could the episodes you watched change a woman's trust about the use of PrEP? (TRUST)
8. Do you think the episodes you watched could make it more likely that a woman will use PrEP?
9. Did the episodes seem too long?
10. Would you want the episodes/series to continue?
11. Did you like the episodes?
12. Could you relate to the characters?
13. Do you think your friends might like to see the episodes?
14. Which of the characters could you relate to the most?

Adapted from Jones, R., Hoover, D. R., & Lacroix, L. J. (2013). A randomized controlled trial of soap opera videos streamed to smartphones to reduce risk of sexually transmitted human immunodeficiency virus (HIV) in young urban African American women. *Nursing outlook*, *61*(4), 205–215.e3. https://doi.org/10.1016/j.outlook.2013.03.006
